# The ABC-X’s of Stress among U.S. Emerging Adults during the COVID-19 Pandemic: Relationship Quality, Financial Distress, and Mental Health

**DOI:** 10.3390/ijerph192013125

**Published:** 2022-10-12

**Authors:** Ashley B. LeBaron-Black, Jeremy B. Yorgason, Melissa A. Curran, Matthew T. Saxey, Rachel M. Okamoto

**Affiliations:** 1 School of Family Life, Brigham Young University, Provo, UT 84602, USA; 2 Department of Family Studies and Human Development, University of Arizona, McClelland Park Rm. 235F, Tucson, AZ 85721, USA

**Keywords:** COVID-19 pandemic, financial distress, mental health, relationship quality, ABC-X Model, moderated mediation, emerging adults, structural equation modeling (SEM)

## Abstract

Many emerging adults have experienced increased financial distress and mental health problems during the COVID-19 pandemic, and isolation may have amplified the importance of close relationships (especially as parents’ influence diminishes during this developmental stage). Using the ABC-X Model to frame our model, we tested whether financial distress (C) mediates the associations between COVID-19 impact (A) and anxiety and depressive symptoms (X), and whether or not romantic relationship quality (B) moderates these indirect associations. Our sample comprised of 1950 U.S. emerging adults in a romantic relationship. Mediation and first-stage moderated mediation were tested using structural equation modeling. Financial distress partially mediated the association between COVID-19 impact and anxiety symptoms and fully mediated the association between COVID-19 impact and depressive symptoms. Strong evidence of moderated mediation was found but in the opposite direction expected: the indirect associations of COVID-19 impact with anxiety and depressive symptoms (through financial distress) were stronger for those in high-quality romantic relationships. The findings may inform policy and practice aimed at optimizing the mental health of emerging adults, especially in light of the ongoing COVID-19 pandemic: specifically, alleviating financial distress may improve the mental health of emerging adults, while focusing on the quality of their romantic relationships may not.

## 1. Introduction

Recent work has documented that the COVID-19 pandemic has impacted the mental health and financial distress of emerging adults [1]. However, scholars have yet to examine (1) if financial distress might explain COVID-19′s impact on the mental health of emerging adults and (2) potential intervention points for emerging adults’ financial distress and mental health as they relate to the impact of COVID-19. Guided by the ABC-X model [2] and with a sample of emerging adults in the USA, we estimated a moderated mediation model to examine these research gaps.

### 1.1. The ABC-X Model

The ABC-X model was a foundational framework for family stress perspectives that have aided research and practice [3,4,5]. One of the reasons the ABC-X model can be a useful perspective is that the application of the theory can identify avenues for intervention [6]. The ABC-X model considers stressors (i.e., events of sufficient magnitude to bring about change; A), resources to cope with a stressor (B), perceptions of a stressor (C), and the resulting level of stress or crisis (X) [2]. In the ABC-X model, resources (B) and perceptions (C) are assumed to have the potential to *moderate* or *mediate* connections between a stressor (A) and the resulting level of stress or crisis (X). We conceptualize the connections between COVID-19 impact, financial distress, relationship quality, and mental health outcomes with these aspects of the ABC-X model in mind.

First, we conceptualize the COVID-19 pandemic as a stressor (A). We identified the COVID-19 pandemic as a stressor because it has been an event of sufficient magnitude to bring about change. One of the changes brought about by the COVID-19 pandemic is an impact on the mental health of emerging adults (i.e., those who are roughly between the ages of 18–30 [7])—specifically, anxiety symptoms [8] and depressive symptoms [9]. Because COVID-19, the stressor, seems to be bringing about a greater level of depressive and anxiety symptoms, we conceptualized these mental health outcomes as the level of stress or crisis resulting from COVID-19 (X).

Next, we considered how resources (B) and perceptions (C) may moderate or mediate the connection between COVID-19 impact and mental health outcomes. One aspect of life the COVID-19 pandemic seems to be highlighting is the importance of finances. The COVID-19 pandemic has brought with it job insecurity and perceptions of financial concern [10]. These perceptions of financial concern—which we refer to as financial distress hereafter—are linked with greater depressive and anxiety symptoms in emerging adults [9,10,11]. Thus, we conceptualize financial distress (C) as a potential *mediator* between COVID-19 impact and mental health outcomes.

**Figure 1:**
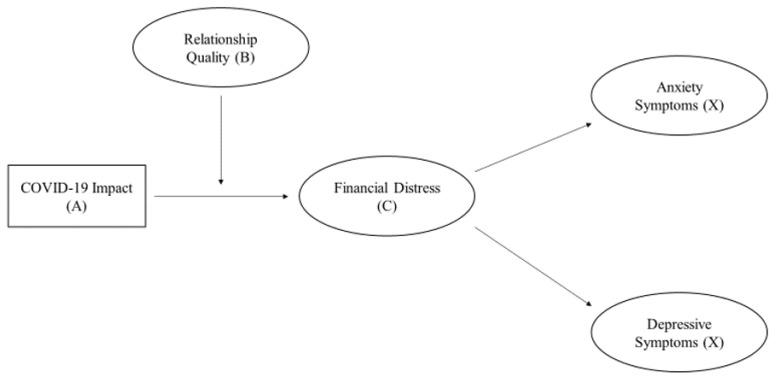
Conceptual Model of First-Stage Moderated Mediation. Note: Ellipses represent latent variables, and the rectangle represents an observed variable. For simplicity, we do not depict controlling for sex, age, and parents’ education level.

What scholars have not yet established is what practices—resources (B)—emerging adults can employ to buffer the negative impact that COVID-19 and financial distress seem to be having on emerging adults’ mental health. Because of a robust negative association between romantic relationship quality and financial distress [12], we suspect that relationship quality (B) may *moderate* the potential *mediation* in our conceptual model (see Figure 1). Cross-sectional mediation models [2,13] and moderated mediation models [14,15] have previously been used to test how resources (B) and perceptions (C) operate to explain the associations between stressors (A) and crisis (X) in applications of the ABC-X model. By conceptualizing our model in this way, we may provide educators and clinicians with evidence to consider in helping emerging adults overcome the negative impacts of COVID-19—and financial distress—on emerging adults’ mental health.

We examine these associations in a sample of emerging adults because finances [16], mental health [17], and romantic relationships [18] are especially salient and impactful during this stage of life. Financial distress may be prevalent in emerging adults due to student loans [16], timing and patterns of financial independence [19], and a lack of basic financial knowledge [20]. If the COVID-19 pandemic increases financial distress [10], it may indirectly impact emerging adults’ depressive and anxiety symptoms more than other populations [9,10,11]. Furthermore, emerging adults may prioritize career pursuits more than romantic relationships [21], and this lack of intentionality in romantic relationships might have implications for coping with this financial distress [12]. At the same time, the influence of romantic relationships in emerging adulthood is magnified by the gradual decline in influence from parents and other family members [22]. Thus, examining these associations in emerging adults seems justified.

### 1.2. COVID-19 Impact and Mental Health

Although many of the preventative measures and responses to COVID-19 have focused on physical protection, there are also emotional and mental consequences that have occurred that likewise deserve intervention [11,23,24]. During the pandemic, there have been widespread increases in depression, anxiety, and stress [1,11]. Containment efforts (e.g., quarantine, social distancing, self-isolation) can impact mental health as individuals feel disconnected from others and disassociated from their “normal” daily life [1,24]. Many have lost loved ones, and the resulting mental health consequences can be compounded in cases where individuals are not able to obtain closure at hospitals or funeral gatherings [25], further implicating the importance of considering mental health during the COVID-19 pandemic.

### 1.3. Financial Distress as a Mediator between COVID-19 Impact and Mental Health

One of the reasons why the COVID-19 pandemic has contributed to higher rates of mental health issues could be because of the pandemic’s effect on financial distress. Financial distress and mental health issues are both extremely prevalent in Western society. Up to 60% of adults ages 21–62 report feeling anxiety when thinking about finances [26] and about one in five adults reportedly suffer from a mental illness [27]. The COVID-19 pandemic may have exacerbated these alarming figures. Individuals have quarantined at home (sometimes resulting in pay disruptions), many have lost their jobs due to shutdowns and other factors, and many parents have been forced to quit their jobs due to a loss of extrafamilial childcare [1,28,29]. During the pandemic, unemployment soared to over 14% [30]; loss of income and employment are linked with increased financial stress [28,31]. In large part due to loss of (or anticipated loss of) work-related income, the onset of COVID-19 was also associated with putting off major purchases, reducing spending on food, increasing credit card debt, and using savings—all of which may serve as indicators of economic hardship [32]. Thus, COVID-19 impact may be positively associated with financial distress due to unemployment and job insecurity [33,34].

The degree to which COVID-19 has had a financial impact relies in part on the economic contexts in which people are situated [1,28,31]. Some evidence suggests that liquid assets may serve as a protective factor between COVID-19 impact and financial distress [34]. In contrast, low-income families may have been disproportionately affected by COVID-19 [32]. Discrimination may also factor into the impact of COVID-19 on individuals because women and minorities may be at greater risk than others to lose their job due to the pandemic [35]. Due to the disparate impact of COVID-19 based on sex, age, and socioeconomic status (SES), we will control for sex, age, and parents’ education level in our analyses. However, this is not a primary focus of the current study. As a whole, the COVID-19 pandemic has certainly increased many individuals’ financial distress.

This financial distress, especially when sustained over longer periods of time, is associated with anxiety [28,36] and depressive symptoms [8,37], as well as lower levels of overall well-being [28,31]. Given the apparent link between COVID-19 impact and higher financial distress, and the well-supported link between financial distress and poor mental health, the impact of COVID-19 on mental health may be mediated by increased financial distress. That is, based on this previous literature, we conceptualized financial distress—a perceptI (C)—as more theoretically meaningful as a mediator than a moderator of this association. Although COVID-19 has been associated with financial distress [1], and financial distress has been associated with depressive and anxiety symptoms [28,37], we do not yet understand if financial distress explains the connections between COVID-19 impact and mental health challenges for emerging adults. Understanding whether and how financial distress explains this association might provide further evidence for where those with influence in helping emerging adults with their mental health might consider focusing. Furthermore, the theoretical meaning of financial distress as a mediator is added to by examining relationship quality as a moderator.

### 1.4. Relationship Quality as a Moderator of the Association between COVID-19 Impact and Financial Distress

Scholars have examined other constructs in the connections between the COVID-19 pandemic, financial distress, and mental health, such as interpersonal relationships and their potential to mitigate the detrimental effects of financial distress. Kelley et al. (2022) found that those who reported increased financial distress due to the COVID-19 pandemic were also likely to report increased relational conflict. However, although financial distress seems to take a toll on relationships [2,12,38], a high-quality relationship may act as a buffer against financial distress and mental health issues [39,40]—engendering the theoretical meaning of relationship quality as a moderator in this study.

For example, Tran et al. (2018) found that in male college students, only those with lower perceived family support exhibited an association between financial strain and anxiety, but those with higher perceived family support seemed to be protected from the negative mental health consequences. Although Tran et al. examined family support as a moderator, we expect to find a similar buffering effect with romantic relationship quality. We expect this because other studies have found that a high-quality relationship buffered directly against mental health issues [41], including one study that specifically examined relationship quality as a moderator in the association between COVID-19 and mental health for married individuals, finding that a high-quality marriage might serve as a buffer [42]. Due to the protective nature of interpersonal relationships for financial distress and mental health [36,43], relationship quality may serve as a resource (B) with which families confront stressors, buffering against negative impact [2]. In essence, because this previous research suggests that relationship quality could buffer the potentially negative impact of COVID-19 on emerging adults’ financial distress *and* depressive and anxiety symptoms, we found greater theoretical meaning in examining relationship quality as a moderator in this study rather than a mediator of the association between COVID-19 impact and emerging adults’ mental health.

### 1.5. The Current Study

Our conceptual model (Figure 1) is conceptualized according to the four facets of the ABC-X model [2] and is based on previous research. Our three hypotheses are:

**H1.**  *COVID-19 impact will be positively associated with financial distress, anxiety symptoms, and depressive symptoms, and financial distress will be positively associated with anxiety and depressive symptoms.*

**H2.**  *Financial distress will mediate the associations of COVID-19 impact with anxiety and depressive symptoms.*

**H3.**  *Romantic relationship quality will moderate the indirect effects of COVID-19 impact on anxiety and depressive symptoms (through financial distress), such that for those in high-quality relationships, the indirect effects will be weaker.*

## 2. Materials and Methods

### 2.1. Participants and Procedure

Our participants were selected from the *Measuring Family Financial Socialization Project* [44]. Data collection, which was funded by the National Endowment for Financial Education, was conducted by Qualtrics Panel during July to September 2020. The project sample was 4182 emerging adults (ages 18–30) living in the USA. Participants were compensated approximately $7.50 for taking a 20-minute online survey. Qualtrics Panel guarantees high-quality data via a “scrubbing” process. Our data was screened for incorrect responses to two attention-check questions, “straight-lining” (i.e., choosing the same response for every question in a block), gibberish in response to open-ended questions, “speeders” (i.e., completing the survey too quickly), duplicate IP addresses, and suspicious or impossible responses (e.g., a credit score above 900). See Qualtrics (2020) for more information on market research panels and participant recruitment. We note that the sample was not nationally representative due to intentional oversampling of minority groups. For the current study, we imposed a filter on the project sample to include only those participants who reported being in a romantic relationship at the time of data collection. Thus, the sample for the current study is 1950 emerging adults.

For marital status, 589 (30.2%) of respondents were married, 6 (0.3%) widowed, 3 (0.2%) separated, 15 (0.8%) divorced, 239 (12.3%) engaged, 1063 (54.5%) “never married”, and 35 (1.8%) preferred not to answer. The average age of participants was 24.76 (SD = 3.68). For sex, 1162 (59.6%) were female and 781 (40.1%) were male, with 2 (0.1%) reporting their sex as “other,” and 5 (0.3%) preferring not to answer. For race/ethnicity, 725 (37.2%) were White, 364 (18.7%) were Black or African American, 402 (20.6%) were Hispanic or Latinx, 236 (12.1%) were Asian, 34 (1.7%) were American Indian or Alaska Native, 16 (0.8%) reported “other,” 158 (8.1%) selected more than one category, and 15 (0.8%) preferred not to answer. Forty-one (2.1%) had attended some high school, 424 (21.7%) had completed high school or equivalent, 526 (27%) had attended some college, 194 (9.9%) had obtained an associate degree, 545 (27.9%) had obtained a bachelor’s degree, 163 (8.4%) had obtained a master’s degree, 52 (2.7%) had completed an advanced degree (i.e., J.D., M.D., Ph.D., etc.), and 5 (0.3%) preferred not to answer. For more information regarding participants and procedure, see LeBaron-Black et al. (2022).

### 2.2. Measures

#### 2.2.1. Independent Variable: COVID-19 Impact

COVID-19 impact was created for the *Measuring Family Financial Socialization Project* [44]. Participants were asked, “How much has the COVID-19 (coronavirus) pandemic affected your life?” Participants responded on a scale of 1 (*Not at all*) to 5 (*Extremely*), with higher scores representing a greater impact. We acknowledge that single-item measures are not as ideal as multi-item scales. However, at the time of data collection (July 2020), we did not find a validated, multi-item scale in the literature that assessed the impact of COVID-19. Additionally, since our sample size is high enough (i.e., over 900), this single-item measure likely performed about as psychometrically well as a multi-item scale [45].

#### 2.2.2. Mediator: Financial Distress

Our measures for financial distress are based on previously developed measures of financial distress [46]. Respondents were asked three questions:, such as “I often worry about my financial situation.” Responses ranged from 1 (*Strongly Disagree*) to 5 (*Strongly Agree*), and higher scores indicate greater financial distress. In our analytical sample, financial distress achieved adequate reliability (α = 0.73).

#### 2.2.3. Moderator: Relationship Quality

Relationship quality is comprised of nine items from Fletcher et al.’s (2000) previous scale development. Participants were asked questions like “How dedicated are you to your relationship?,” “How much do you trust your partner?,” and “How happy are you with your relationship?” Response options ranged from 1 (*Not at all*) to 7 (*Extremely*). Higher scores indicate a higher quality romantic relationship. In our analytical sample, relationship quality achieved sound reliability (α = 0.95).

#### 2.2.4. Dependent Variables: Anxiety and Depressive Symptoms

Our measures for anxiety symptoms are based on previously developed anxiety measures [47]. Participants were shown seven statements such as “I feel restless, as if I have to be on the move” and “I feel tense or ‘wound up.’ ” Response options included ranges such as from 1 (*Not at all*) to 4 (*Most of the time*) and from 1 (*Not at all*) to 4 (*Very definitely and quite badly*). Regardless of the exact wording, higher scores represented greater anxiety. One item was reverse coded to ensure higher scores indicated greater anxiety. After this reverse coding, anxiety symptoms achieved adequate reliability (α = 0.80).

Our measures for depressive symptoms are also based on Zigmond and Snaith’s (1983) measures for depression. Participants were shown seven statements such as “I feel as if I am slowed down” and “I have lost interest in my appearance.” Response options included ranges such as 1 (*Not at all*) to 4 (*Nearly all the time*). Irrespective of the specific wording of responses, higher scores suggested greater depression. Five items were reverse coded to ensure that higher scores indicated higher levels of depression. Following this reverse coding, depressive symptoms achieved acceptable reliability (α = 0.76).

#### 2.2.5. Control Variables: Sex, Age, and Parents’ Education Level

Participants were asked, “What is your sex (assigned at birth)?” Responses were coded as 0 = female (*n* = 1,162) and 1 = male (*n* = 781), with “Other” and “Prefer not to answer” coded as missing (*n* = 7). Age in years was measured continuously from 18 to 30, with one participant coded as missing. Participants were asked, “What is the highest level of education either of your parents has received?” Responses were coded into a binary variable where 0 = no college degree (less than high school, high school or equivalent, some college; *n* = 936) and 1 = college degree (associate, bachelor’s, master’s, advanced degree; *n* = 999), with “Prefer not to answer” coded as missing (*n* = 15).

#### 2.2.6. Data Analysis Plan

First, we conducted preliminary analyses in SPSS to determine descriptive statistics and bivariate correlations among study variables. Three models were estimated in Mplus Version 8, all adjusted for participants’ sex, age, and parents’ education level. There were no missing data on any of our main study variables; missing data on control variables ranged from 0.1 to 0.8%. This missing data were handled using full information maximum likelihood (FIML). Comparative fit index (CFI), root mean square error of approximation (RMSEA), and standardized root mean-square residual (SRMR) were used to determine absolute model fit [48]. The following indicated “good” model fit: CFI > 0.95, RMSEA < 0.05, SRMR < 0.05 (Little, 2013). To determine effect size of standardized direct effects, cutoffs from Cohen (1988) were used: 0.1 = small, 0.3 = medium, and 0.5 = large. To determine effect size of standardized indirect effects, cutoffs from Kenny (2021) were used: 0.01 = small, 0.09 = medium, and 0.25 = large.

In Model 1, we estimated a structural equation model (SEM) to test the direct effects of COVID-19 impact on financial distress, anxiety symptoms, and depressive symptoms and the direct effects of financial distress on anxiety and depressive symptoms, as well as the indirect effects of COVID-19 impact on anxiety and depressive symptoms through financial distress. Anxiety and depressive symptoms were correlated with each other. Standardized coefficients and 95% confidence intervals were estimated for indirect associations between COVID-19 impact and anxiety and depressive symptoms (through financial distress) using 5000 bootstraps. SEM allowed us to utilize latent variables, reducing measurement error [49]. We used the latent variance method so that each latent variable was standardized and its factor loadings could be freely estimated [50]. Modification indices were used to make minor adjustments to the model that improved model fit (e.g., error correlations between an individual anxiety item and an individual depression item).

Next, we estimated two moderated mediation models (also with 5000 bootstraps) to test whether romantic relationship quality moderated the indirect effect of COVID-19 impact (through financial distress) on anxiety symptoms (Model 2) and depressive symptoms (Model 3); the two models were identical besides the differing outcome variable. Specifically, we estimated a first-stage moderated mediation model [48] (see Model 8 in Stride et al., 2015), where the first stage of the indirect effect (i.e., the pathway from COVID-19 impact to financial distress) was moderated by the value of romantic relationship quality. In this way, we explored if the direct effect of COVID-19 impact on financial distress changed over levels of relationship quality, and if the indirect effect of COVID-19 on anxiety/depressive symptoms (through financial distress) changed over levels of relationship quality. Using mean scales in place of latent variables, we created an interaction term using COVID-19 impact and relationship quality. Regression paths were then estimated from COVID-19 impact, relationship quality, and financial distress onto anxiety/depressive symptoms as well as from COVID-19 impact, relationship quality, and the interaction term onto financial distress. COVID-19 impact, relationship quality, and the interaction term were all correlated with each other. We then used the model constraint subcommand to test simple slopes of the conditional indirect effects with low (i.e., one standard deviation below the mean), medium (i.e., the mean), and high (i.e., the highest value) values of romantic relationship quality. We used loop plots to plot the conditional indirect associations between COVID-19 impact and anxiety/depressive symptoms (through financial distress) for low, medium, and high values of relationship quality. Finally, we conducted three post hoc Wald tests each for Model 2 and Model 3 to determine whether the indirect effects at low versus medium, medium versus high, and low versus high levels of relationship quality differed significantly from each other.

## 3. Results

### 3.1. Preliminary Analyses

Descriptive statistics and bivariate correlations are in Table 1. Many emerging adults in our sample have been at least somewhat affected by COVID-19 (M = 3.41 out of 5; SD = 1.11). Furthermore, some participants reported experiencing financial distress (M= 2.80 out of 5; SD = 1.08). Likewise, some participants reported experiencing anxiety symptoms (M = 2.30 out of 4; SD = 0.67) and depressive symptoms (M = 1.81 out of 4; SD = 0.56). In terms of relationship quality, participants reported relatively high-quality romantic relationships (M = 6.09 out of 7; SD = 1.13). All bivariate correlations among the main study variables were in the expected directions and significant at the *p* < 0.001 level, with the exception of the correlation between COVID-19 impact and relationship quality, which was not statistically different from zero.

### 3.2. Structural Models

Standardized direct effects from Models 1, 2, and 3 can be seen in Table 2.

**H1.**  *COVID-19 impact will be positively associated with financial distress, anxiety symptoms, and depressive symptoms, and financial distress will be positively associated with anxiety and depressive symptoms.*

Model 1 fit the data appropriately: CFI = 0.95; RMSEA = 0.04 with 90% Confidence Interval [0.038, 0.045]; SRMR = 0.03. The model explained 16% of the variance in financial distress, 41% of the variance in anxiety symptoms, and 39% of the variance in depressive symptoms. As seen in Table 2, COVID-19 impact was positively associated with financial distress (*β* = 0.37, *p* < 0.001; between medium and large effect size) and anxiety symptoms (*β* = 0.07, *p* = 0.01; very small effect size) but was not significantly associated with depressive symptoms (*β* = −0.03, *p* = 0.27). Financial distress was positively associated with both anxiety (*β* = 0.58, *p* < 0.001; large effect size) and depressive symptoms (*β* = 0.63, *p* < 0.001; large effect size). Because on a bivariate level COVID-19 impact was negatively associated with depressive symptoms (see Table 1), we consider H1 to be fully supported, with the lack of direct association in the SEM likely due to full mediation, as presented next.

**H2.**  *Financial distress will mediate the associations of COVID-19 impact with anxiety and depressive symptoms.*

In terms of standardized indirect effects from Model 1, COVID-19 impact was indirectly associated with both anxiety symptoms (*β* = 0.22, *p* < 0.001, 95% CI [0.18, 0.26]; about a large indirect effect size) and depressive symptoms (*β* = 0.23, *p* < 0.001, 95% CI [0.19, 0.28]; about a large indirect effect size) through financial distress. Given that a significant direct association between COVID-19 impact and anxiety symptoms remained, the indirect effect indicates partial mediation of this path. On the other hand, because there was no significant direct association between COVID-19 impact and depressive symptoms (but the bivariate association was significant), the indirect effect indicates full mediation of this path. Thus, H2 was fully supported.

**H3.**  *Romantic relationship quality will moderate the indirect effects of COVID-19 impact on anxiety and depressive symptoms (through financial distress), such that for those in high-quality relationships, the indirect effects will be weaker.*

Model 2 fit the data appropriately: CFI = 0.998; RMSEA = 0.03 with 90% Confidence Interval [0.000, 0.076]; SRMR = 0.001. Model 2 explained 14% of the variance in financial distress and 27% of the variance in anxiety symptoms. Model 3 also had good model fit: CFI = 1.00; RMSEA = 0.00 with 90% Confidence Interval [0.000, 0.035]; SRMR = 0.00. Model 3 explained 14% of the variance in financial distress and 29% of the variance in depressive symptoms.

**Figure 2:**
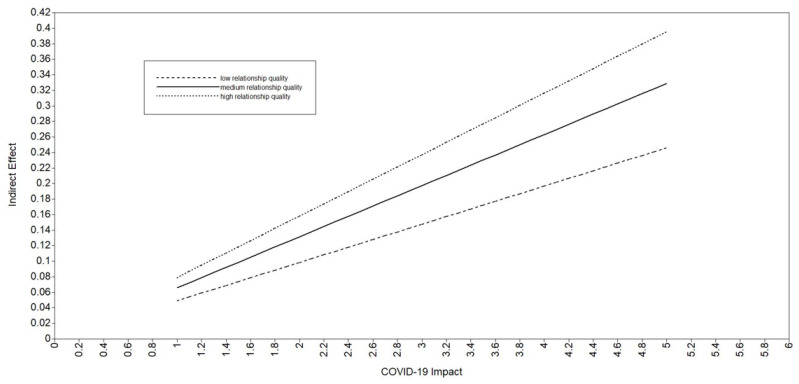
Plot of the Indirect Effect of COVID-19 Impact on Anxiety Symptoms Through Financial Distress at Different Levels of Romantic Relationship Quality (Model 2). Note: low = one standard deviation below the mean, medium = the mean, high = the highest value.

**Figure 3:**
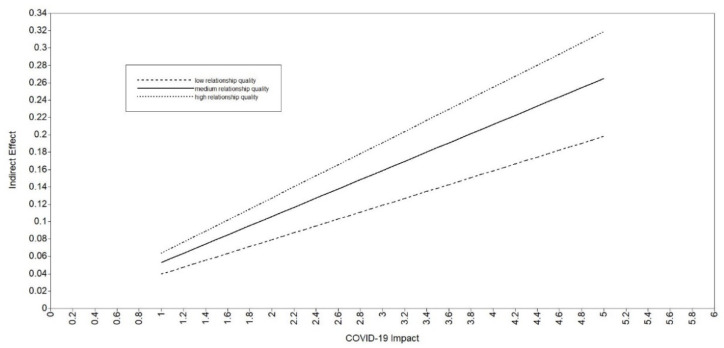
Plot of the Indirect Effect of COVID-19 Impact on Depressive Symptoms Through Financial Distress at Different Levels of Romantic Relationship Quality (Model 3). Note: low = one standard deviation below the mean, medium = the mean, high = the highest value.

As per Table 2, the interaction term (i.e., COVID-19 impact * relationship quality) was significantly associated with financial distress both in Model 2 and in Model 3 (*β* = 0.48, *p* = 0.001 in both models), providing evidence of first-stage moderated mediation. As further evidence that COVID-19 impact is associated with anxiety and depressive symptoms through financial distress at different levels of relationship quality, tests of simple slopes were significant (*p* < 0.001) at low, medium, and high values of relationship quality in Model 2 (low: b = 0.05; medium: b = 0.07; high: b = 0.08) and in Model 3 (low: b = 0.04; medium: b = 0.05; high: b = 0.06). For Model 2, three post hoc Wald tests indicated that the indirect effect of COVID-19 impact on anxiety symptoms (through financial distress) differed between the following levels of relationship quality: low versus medium (*p* < 0.001), medium versus high (*p* < 0.001), and low versus high (*p* < 0.001). Similarly, for Model 3, the Wald tests indicated that the indirect effect of COVID-19 impact on depressive symptoms (through financial distress) differed between the following levels of relationship quality: low versus medium (*p* < 0.001), medium versus high (*p* < 0.001), and low versus high (*p* < 0.001). In sum, the indirect associations of COVID-19 impact with anxiety and depressive symptoms through financial distress were stronger as levels of relationship quality increased. Connecting back to H3, the findings of the moderated mediation were opposite of the hypothesis: the indirect effects of COVID-19 impact on anxiety and depressive symptoms (through financial distress) were stronger for those in high-quality romantic relationships. The plots seen in Figure 2 and Figure 3 provide a visual for the conditional indirect effect of COVID-19 impact on anxiety/depressive symptoms (through financial distress) at low, medium, and high values of relationship quality. Together, these results provide strong evidence of moderated mediation, albeit opposite of what was hypothesized for H3.

## 4. Discussion

Guided by the ABC-X model, in a sample of emerging adults who were relatively diverse in race, ethnicity, and education, we tested (1) if financial distress mediates the associations between COVID-19 impact and anxiety and depressive symptoms and (2) if romantic relationship quality moderates these indirect effects. We focused on emerging adults as finances, mental health, and romantic relationships are often salient constructs during this stage of life [16,17,18].

In support of the ABC-X model and as hypothesized (H1), we found that COVID-19 impact was positively associated with financial distress, anxiety symptoms, and depressive symptoms, and financial distress was positively associated with anxiety and depressive symptoms. Evidence for this hypothesis comes from the bivariate correlations that we tested and the direct associations from the SEM. Most of these direct effects are considered medium to large [51]. This finding for the current study is line with previous literature, in which individuals affected by the COVID-19 pandemic have experienced high levels of financial distress [28] and mental health issues [52]. Furthermore, in support of the ABC-X model and as hypothesized (H2), we found that financial distress mediates the associations of COVID-19 impact with anxiety and depressive symptoms—both with large effect sizes [53]. Per the ABC-X model [2], perceptions (i.e., financial distress) can mediate associations between stressors (i.e., COVID-19 impact) and level of crisis (i.e., anxiety and depressive symptoms). These findings align with previous research findings linking financial distress to anxiety [28,36] and depressive symptoms [8,37]. The mediation finding from the current study is important in providing an explanation for why we have seen an increase in mental health issues during the pandemic among emerging adults. Collectively, these findings emphasize the importance of optimizing the mental health of emerging adults given the impact of COVID-19 while offering a focal point for analysis and policy, which is specific to alleviating financial stress for these emerging adults.

In comparison to the first two findings just discussed, the moderated mediation results were surprising. Although we found strong evidence that romantic relationship quality moderated the indirect associations between COVID-19 impact and anxiety and depressive symptoms (through financial distress), the results were the opposite of what we had hypothesized: the indirect associations were *stronger* as levels of relationship quality increased. This is surprising given previous research findings that high-quality relationships buffer against financial distress and mental health issues [36,41,42]. Linking this previous research to the ABC-X model [2], we expected relationship quality to be a resource buffering (i.e., moderating) the negative effects.

We discuss some ideas for why we might have found this moderation, opposite of the hypothesis. First, although the indirect effect was significant at all levels of relationship quality (in the same direction), all three levels of romantic relationship quality we tested (i.e., high, medium, and low) were actually relatively high levels of romantic relationship quality due to the skewed nature of this variable. The “high” level was the highest possible score (7.0), the “medium” level was the mean (6.09), and the “low” level was one standard deviation below the mean (4.96 on a 1–7 Likert scale). Thus, “low” levels of relationship quality in the current study were quite high. This is to be expected, as mean reports of relationship quality tend to be high in most samples [54]. It is possible that relationship quality does serve as a buffer but only for certain types of relationships. Relationship quality may be especially salient among older married individuals; Umberson and Thomeer (2020) found that marital quality is more salient for constructs such as health at older ages than at younger ages. Future research could test the pathways of relationship quality for individuals who share children together but who may not be romantically involved with one another [55].

Second, and as connected to the ABC-X model, it may be that resources other than relationship quality are salient to individuals in romantic relationships during a pandemic. For example, based on 29 articles specific to relationships during COVID-19, Lannutti and Bevan (2022) discussed that in several of the studies from the Special Issue, dyadic coping between partners (defined as one partner communicating their stress to their partner and the second partner evaluating and responding in turn, for example with empathy) was a resource for individuals in couple relationships during the pandemic. To this same point, the COVID-19 pandemic may have offered individuals in romantic relationships opportunities to reflect and reframe in their relationships, including individuals reporting on how their relationship had changed in terms of sharing responsibilities, togetherness, and space because of the pandemic [56].

Third, the data are cross-sectional, and the directionality of pathways is not clear. Thus, perhaps couples who experienced high levels of financial distress and/or mental health issues together grew closer together and thus reported higher relationship quality. Previous research has found that for some couples, experiencing stressors together can improve the quality and commitment in their relationship [44,57]. It is possible that our results indicate not that lower-quality relationships worsen the negative effects of COVID-19 impact, but rather that those who have experienced the most negative effects of COVID-19 impact have simultaneously experienced an increase in relationship quality. In future research, longitudinal and cross-lagged models would help determine directionality of associations. However, because highly satisfied couples are more likely to be highly interdependent [58], it could be that mental distress regarding finances is exacerbated by worry about the potential negative relational impact of the financial strain.

In terms of implications, our findings may inform policy (e.g., by government, colleges and universities, healthcare institutions, workplaces) and practice (e.g., by mental health professionals, financial counselors and planners, financial therapists, social workers, healthcare providers, college and career advisors) aimed at optimizing the mental health of emerging adults, especially in light of the ongoing COVID-19 pandemic. Specifically, alleviating financial distress may improve the mental health of emerging adults, while focusing on the quality of their romantic relationships may not. Recentered efforts to focus on constructs such as financial distress for emerging adults may be useful to researchers who are using theories such as the ABC-X model to understand more about stressors (A), resources (B), perceptions (C; e.g., financial distress), and resulting levels of stress or crisis (X). For example, Kelley et al. (2022) used the family adjustment and adaptation response model (which built on the ABC-X model [4]) as a framework to study the relational impact of changes in financial stress during the COVID-19 pandemic. They found that while decreased financial stress was associated with decreased conflict (and increased financial stress was associated with increased conflict), decreased financial stress was also associated with decreased emotional closeness and relationship happiness. Thus, various aspects of relationship quality seem to be linked to financial distress in complicated ways. Further, the COVID-19 pandemic altered the lives of many emerging adults, leading to potential loss of routine, lack of social contact, and changes in work and finances [11], which may have drawn the focus for emerging adults from the quality of their romantic relationships to the quality of their mental health. It may be that the timing of data collection during the COVID-19 pandemic for these study constructs also made a difference, as others have found that when assessed over time, depression (but not anxiety) increased over time due to feelings of loneliness [59].

As for limitations and future research directions, we note our use of cross-sectional data to study mediation and moderated mediation. Although other studies have used an ABC-X lens to test cross-sectional mediation [2,13] and moderated mediation [14,15], to determine directionality in mediating analyses longitudinal data is needed [60]. Thus, our results should be interpreted with caution because the directionality of the pathways we tested was solely informed by theory and previous research. Perhaps longitudinal data that captures *change in* romantic relationship quality might help determine what moderating role relationship quality plays in the associations tested. Another limitation is that our stressor (i.e., COVID-19 impact) was not objectively measured; the measure we used was self-report and therefore could arguably be classified as a perception. Additionally, we acknowledge the disparate impact of the COVID-19 pandemic by sex, race, and SES [32,35]. Although these factors were not explored in depth in the current study, future research could use additional theories and lenses to determine whether the findings of the current study might differ depending on individuals’ and couples’ demographic characteristics (e.g., race, gender, class) and to explore other potential buffers between COVID-19 impact and mental health for different groups. Our study was limited in that we did not have access to some potentially relevant control variables (e.g., physical health, social support from those other than romantic partners, and income). Finally, our study was specific to emerging adults in the USA. Although two studies during the COVID-19 pandemic in Southeast Asia [61] and Australia [62] have found similar negative associations between financial distress/perceived financial status and mental health in samples that included emerging adults, more research outside the USA among emerging adults, specifically, is needed to generalize our findings outside the USA. For example, a country-by-country comparison of our findings among emerging adults would be an important area for future research to understand if a similar phenomenon is emerging in places such as Europe, Asia, South America, and Africa. As others have argued [22,63], a focus on samples outside the USA specific to finances is still needed.

## 5. Conclusions

In conclusion, emerging adults (who are already at increased risk of financial and mental health issues [16,17] have been highly impacted by the COVID-19 pandemic. We found evidence that this reported impact is positively associated with anxiety and depressive symptoms, and our study provided novel insights into the mechanisms at play and how these negative effects might be avoided or alleviated. Our findings suggest that financial distress accounts for much of this link between the pandemic and emerging adults’ mental health. That is, those emerging adults who have been highly impacted by the pandemic are likely to be experiencing mental health issues because they are experiencing high levels of financial distress. Further, we found that a high-quality romantic relationship did not buffer against these negative effects—in fact, those in the highest-quality relationships had the strongest indirect effects. Thus, in efforts to improve the mental health of emerging adults, focusing on their relationships may not be effective; instead, relieving emerging adults’ financial distress may lessen the negative impact of the pandemic.

## Figures and Tables

**Table 1:** Descriptive statistics and bivariate correlations (N = 1950).

Variables	1	2	3	4	5	6	7	8
1. COVID-19 Impact	--							
2. Relationship Quality ^a^	−0.02	--						
3. Financial Distress ^a^	0.31 ***	−0.18 ***	--					
4. Anxiety Symptoms ^a^	0.28 ***	−0.22 ***	0.44 ***	--				
5. Depressive Symptoms ^a^	0.17 ***	−0.39 ***	0.43 ***	0.59 ***	--			
6. Sex ^b, c^	−0.08 ***	−0.05 *	−0.05 *	−0.14 ***	−0.03	--		
7. Age	−0.08 ***	−0.02	−0.06 **	−0.14 ***	−0.06 *	0.08 ***	--	
8. Parents’ Education ^b, d^	0.00	0.08 ***	−0.07 **	−0.10 ***	−0.12 ***	0.10 ***	0.13 ***	--
Mean or %	3.41	6.09	2.80	2.30	1.81	40.1%	24.76	51.2%
Standard Deviation	1.11	1.13	1.08	0.67	0.56	--	3.68	--
Range	1–5	1–7	1–5	1–4	1–4	0–1	18–30	0–1

Note: Statistically significant correlations are bolded. ^a^ Mean scores were created for scales. ^b^ % represents the amount of participants included in the category represented as 1. ^c^ Comparison group is female. ^d^ Comparison group is no college degree. * *p* < 0.05. ** *p* < 0.01. *** *p* < 0.001.

**Table 2:** Standardized direct associations.

	Financial Distress			Anxiety Symptoms			Depressive Symptoms		
	Model 1	Model 2	Model 3	Model 1	Model 2	Model 3	Model 1	Model 2	Model 3
COVID-19 Impact	0.37 ***	0.11	0.11	0.07 **	0.15 ***	--	−0.03	--	0.05 *
Financial Distress	--	--	--	0.58 ***	0.36 ***	--	0.63 ***	--	0.35 ***
Relationship Quality	--	−0.41 ***	−0.41 ***	--	−0.16 ***	--	--	--	−0.32 ***
COVID-19 Impact x Relationship Quality	--	0.48 **	0.48 **	--	--	--	--	--	--
Sex ^a^	−0.04	−0.02	−0.02	−0.09 ***	−0.10 ***	--	0.01	--	−0.02
Age	−0.04	−0.03	−0.03	−0.09 ***	−0.09 ***	--	−0.01	--	−0.03
Parents’ Education ^b^	−0.09 **	−0.05 *	−0.05 *	−0.05	−0.04	--	−0.05 *	--	−0.06 **

Note: Statistically significant associations are bolded. ^a^ Comparison group is female. ^b^ Comparison group is no college degree. * *p* < 0.05. ** *p* < 0.01. *** *p* < 0.001.

## Data Availability

The data used in the current study are not publicly available due to privacy restrictions set forth in the participant consent form and by the institutional review board. However, the analysis code/syntax used in the current study is available upon request from the first author.
